# Illuminating mitochondrial translation through mouse models

**DOI:** 10.1093/hmg/ddae020

**Published:** 2024-05-23

**Authors:** Laetitia A Hughes, Oliver Rackham, Aleksandra Filipovska

**Affiliations:** Telethon Kids Institute, Northern Entrance, Perth Children’s Hospital, 15 Hospital Avenue, Nedlands, WA 6009, Australia; Harry Perkins Institute of Medical Research, 6 Verdun Street, Nedlands, WA 6009, Australia; ARC Centre of Excellence in Synthetic Biology, 35 Stirling Highway, Crawley, WA 6009, The University of Western Australia, Crawley, WA 6009, Australia; Telethon Kids Institute, Northern Entrance, Perth Children’s Hospital, 15 Hospital Avenue, Nedlands, WA 6009, Australia; Harry Perkins Institute of Medical Research, 6 Verdun Street, Nedlands, WA 6009, Australia; ARC Centre of Excellence in Synthetic Biology, 35 Stirling Highway, Crawley, WA 6009, The University of Western Australia, Crawley, WA 6009, Australia; Curtin Medical School, Curtin University, Kent Street, Bentley, WA 6102, Australia; Curtin Health Innovation Research Institute, Curtin University, Kent Street, Bentley, WA 6102, Australia; Telethon Kids Institute, Northern Entrance, Perth Children’s Hospital, 15 Hospital Avenue, Nedlands, WA 6009, Australia; ARC Centre of Excellence in Synthetic Biology, 35 Stirling Highway, Crawley, WA 6009, The University of Western Australia, Crawley, WA 6009, Australia; Department of Biochemistry and Molecular Biology, Monash Biomedicine Discovery Institute, Monash University, 19 Innovation Walk, Clayton, Clayton, VIC 3168, Australia

**Keywords:** mitochondria, gene expression, protein synthesis, animal models

## Abstract

Mitochondria are hubs of metabolic activity with a major role in ATP conversion by oxidative phosphorylation (OXPHOS). The mammalian mitochondrial genome encodes 11 mRNAs encoding 13 OXPHOS proteins along with 2 rRNAs and 22 tRNAs, that facilitate their translation on mitoribosomes. Maintaining the internal production of core OXPHOS subunits requires modulation of the mitochondrial capacity to match the cellular requirements and correct insertion of particularly hydrophobic proteins into the inner mitochondrial membrane. The mitochondrial translation system is essential for energy production and defects result in severe, phenotypically diverse diseases, including mitochondrial diseases that typically affect postmitotic tissues with high metabolic demands. Understanding the complex mechanisms that underlie the pathologies of diseases involving impaired mitochondrial translation is key to tailoring specific treatments and effectively targeting the affected organs. Disease mutations have provided a fundamental, yet limited, understanding of mitochondrial protein synthesis, since effective modification of the mitochondrial genome has proven challenging. However, advances in next generation sequencing, cryoelectron microscopy, and multi-omic technologies have revealed unexpected and unusual features of the mitochondrial protein synthesis machinery in the last decade. Genome editing tools have generated unique models that have accelerated our mechanistic understanding of mitochondrial translation and its physiological importance. Here we review the most recent mouse models of disease pathogenesis caused by defects in mitochondrial protein synthesis and discuss their value for preclinical research and therapeutic development.

## Introduction

Mitochondria produce more than 90% of the ATP required by our bodies via oxidative phosphorylation (OXPHOS) and thereby have a fundamental role in cell and energy metabolism [1]. Mitochondria are composed of proteins encoded by both the nuclear and mitochondrial genomes and the coordinated expression of both genomes is essential for energy production [2]. Impaired energy production leads to mitochondrial dysfunction that causes or contributes significantly to diverse diseases including mitochondrial diseases [3, 4]. Mitochondrial pathologies result from mutations or variations in nuclear or mitochondrial genes that encode proteins or regulatory RNAs essential for mitochondrial biogenesis [1, 3, 4]. Uncoordinated mitochondrial and nuclear gene expression causes mitochondrial dysfunction and compromised energy production in mitochondrial diseases and in other common diseases [1, 3, 4]. Mitochondrial diseases affect approximately 1 in 5000 live births [5, 6], furthermore, the prevalence of oligosymptomatic carriers of mt-DNA mutation is as high as 1 in 200 [7]. Mutations in components of the translation machinery, including mitoribosomal proteins, regulatory factors, and auxiliary translation factors, collectively cause the majority of mitochondrial diseases*.* Biochemically these disorders can manifest as either isolated or combined OXPHOS complex deficiency causing multi-systemic disorders [3, 4]. The pathomechanisms underlying these diseases have most frequently been investigated in patient cells or cell models [1], however, improved gene editing technologies have accelerated the production and availability of *in vivo* models.

Animals provide many advantages over cell models of disease, which rely on glycolytic and high oxygen conditions that are not physiologically accurate. *In vivo* models provide not only physiologically relevant conditions, but the availability of target tissues implicated in the pathologies. The mouse is the pre-eminent model used to study human diseases, due to genome homology, anatomical similarity, and effective gene editing tools available to manipulate the nuclear genome. With the availability of CRISPR/Cas9 editing, it is now possible to generate complex genetic mouse lines including knockout, knockdown and knock-in mutations, to be studied in homozygous, heterozygous, tissue-specific and drug-induced models. These models have been essential in establishing our current understanding of mitochondrial translation and associated diseases.

## Critical and unique roles of mitoribosomal proteins in translation

Mammalian mitochondrial ribosomes are specialized molecular machines that recognize the unique structures of mitochondrial mRNAs to co-translationally insert the highly hydrophobic *de novo* synthesized polypeptides into the inner membrane for OXPHOS assembly. The 55S mitoribosome consists of a 28S small subunit composed of the 12S rRNA and 30 proteins, and the 39S large subunit contains of the 16S rRNA and 52 proteins [8]. Mammalian mitoribosomes lack a 5S rRNA found in bacterial ribosomes, which has been replaced by a mitochondrial tRNA [9–11]. Mitoribosomes have acquired a higher protein to rRNA ratio during evolution compared to bacterial and cytoplasmic ribosomes [8, 12, 13] and 36 of these new proteins are mitochondria-specific [8]. Although the role of the additional protein complement is not known, it is possible that the accessory proteins facilitate the recognition of the unconventional features of mitochondrial mRNAs, including the absence of 5′-7-methylguanosine cap structures and 5′- and 3′-untranslated regions, as well as the use of non-canonical start and stop codons. For example, during translation initiation, the mitochondrial ribosomal protein of the small subunit 39 (MRPS39/PTCD3) assists with mRNA recognition and docking at the mRNA entrance site of the small subunit [14–16], and the mitochondria-specific MRPS5 protein extension lines the entry channel and guides the mRNA toward the P-site for start codon recognition [16]. At the large subunit, the N-terminal extension of the mitochondria-specific MRPL45 protein extends into the exit tunnel to direct the progression of the nascent chain and prevent helix formation [16, 17]. To facilitate co-translational inner membrane insertion of hydrophobic proteins, the mitoribosome associates with the inner mitochondrial membrane through contacts between MRPL45, MRPL28, MRPL29 and MRPL24 with the inner membrane protein, OXA1L [16, 17]. These interactions are facilitated by the mitochondria specific phospholipid, cardiolipin [18], which structurally supports the inner membrane cristae [19]. Supernumerary mitoribosomal proteins lack functional redundancy and show little to no amino acid sequence homology between each other [20]. There are more than 80 mammalian mitoribosome proteins, most of which are essential and robustly expressed by embryonic day 7.5 in mice [20].

Pathogenic mutations in the 12S rRNA, associated with maternally transmitted deafness [21, 22] impair tRNA loading in the A-binding site in the decoding center, resulting in reduced translational accuracy [23]. Since introducing mutations in the mitochondrial genome is not yet effective, the physiological effect of conserved mitoribosomal proteins on translation fidelity has been investigated in several mouse models by mutating amino acids located in the decoding site of the small ribosomal subunit. A homozygous V338Y mutation in the highly conserved MRPS5 protein, nested at the small ribosomal subunit entry channel [12], causes stress-induced behavioral changes and hearing loss in mice, mimicking similar pathologies found in patients [24]. Similarly, introducing mutations in neighboring residues within the mouse MRPS12 protein can result in error-prone or hyper-accurate translation in mice [25]. Error-prone translation caused amino acid misincorporation into newly synthesized mitochondrially-encoded proteins, impairing their stability that triggered a transcriptional stress response stimulating mitochondrial biogenesis and liver regeneration by increasing cell proliferation [25]. In contrast, hyperaccurate mitochondrial translation results in slower rate of translation that was incompatible with the requirements of high energy demand, post mitotic tissues, resulting in cardiomyopathy [25, 26], that models the phenotype observed in patients with mutations in mitochondrial ribosomal proteins [27–31]. Hyperaccurate translation does not induce a stress response like the error-prone, resulting in permanent decline and progressive pathologies in these mice. Remarkably, the timely activation of stress response pathways has been demonstrated to restore mitochondrial function in patients with disrupted translation causing reversible infantile respiratory chain deficiency (RIRCD) [32]. This disease presents as a severe metabolic disturbance in muscle, leading to hypotonia and weakness that spontaneously resolves after 6 months of age [32]. Studying this disease from the onset of metabolic crisis to recovery, reveals an initial, transient induction of the integrated stress response, followed by mTOR activation and metabolic shift to TCA and fatty acid oxidation, to enhance mitochondrial biogenesis. The transient nature of the integrated stress response is particularly important for recovery, as chronic upregulation of this pathway has been shown to be detrimental to muscle function [33]. Interestingly, the activation of stress response pathways induced by cardiac and skeletal muscle-specific loss of the aspartyl-tRNA synthetase (DARS2) in mice was tissue specific and independent of respiratory chain deficiency [34]. Notably, while protein synthesis and respiratory chain function was comparably disrupted in both tissues, only cardiomyocytes initiated stress response pathways, while the skeletal muscle has a greater intrinsic proteostatic buffering capacity preventing unassembled protein accumulation, which would otherwise trigger stress response pathways [34]. These studies highlight the particularly strong influence of tissue-specific stress responses in mediating the presentation of diseases caused by defects in mitochondrial translation.

Environmental factors, such as a high-fat diet in mice, can compound the effects of the error-prone or hyperaccurate mutations in MRPS12 [26]. While low level mistranslation is beneficial to liver function under a normal diet [25], a high-fat diet induced additional stress that caused liver disease and activated steroid and amino acid metabolism [26]. Interestingly, mistranslation conferred an advantage for heart function on a high-fat diet, suggesting that postmitotic and highly proliferative tissues possess different stress buffering capacities. The effect of external stress on disease severity has been documented in case reports of patients with mutations affecting ribosomal proteins such as MRPL44 [35], and MRPL12 [36] and in translation factors such as the mitochondrial elongation factor Tu (TUFM) [37], where unrelated infections exacerbated disease pathologies. In a study of fibroblasts isolated from a patient with a *TUFM* mutation, under basal conditions ROS levels were not different to controls, however upon exposure to oxidative stress ROS production was significantly increased, hence their stress buffering capacity is clearly diminished [38]. Recently, TUFM has been implicated in mitophagy upon viral infection, by localizing in part to the outer mitochondrial membrane [39], which could contribute to the reduced capacity of patient fibroblasts to cope with exposure to stress. Although external factors can significantly modulate disease presentation, it is important to consider any additional roles of translation factors in their contribution to diseases pathologies that may be independent of effects on protein synthesis.

In addition to environmental stress, other factors such as the mitochondrial inner membrane lipid composition or the insertase OXA1 can affect mitoribosome association and co-translational OXPHOS assembly [14, 17, 18]. The most abundant phospholipid in the mitochondrial inner membrane, cardiolipin, was shown to be required for mitoribosome docking and translation [18]. Pathogenic mutations in the cardiolipin synthesis enzyme (CRLS1) lead to loss of cardiolipin, which reduces the association of the mitoribosome with the inner membrane and resulted in decreased translation [40]. Similarly, pathogenic mutations in OXA1L cause significant reduction in translation that leads to severe mitochondrial disease [41].

## Modeling the role of mitochondrial RNA regulators in disease

**Figure 1:**
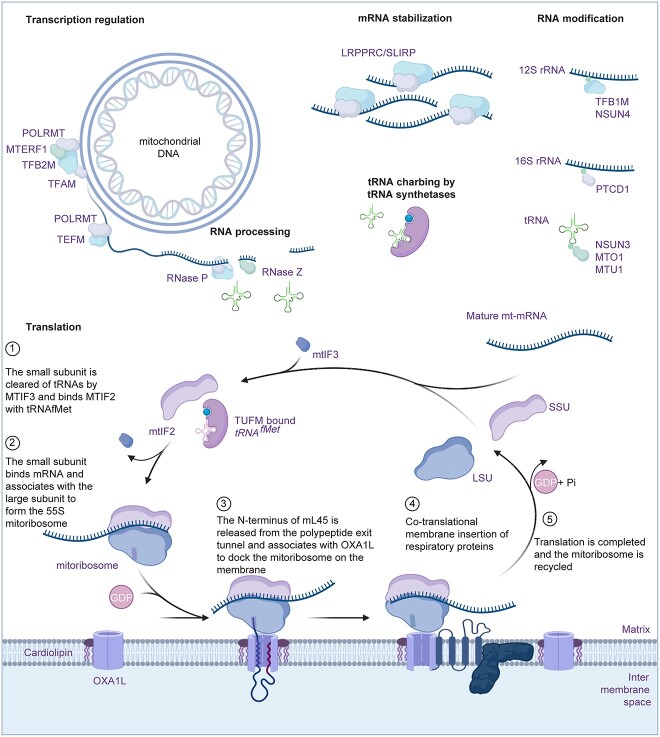
Regulation of mitochondrial gene expression. Translation of mtDNA-encoded proteins is regulated by many nuclear encoded factors. The mitochondrial genome is transcribed as a near genome-length, polycistronic transcripts by POLRMT and TEFM, which are processed by the RNase P complex (MRPP1, MRPP2, MRPP3) and the RNase Z (ELAC2) endonuclease to release individual mt-RNAs. A diverse range of enzymes modify, stabilize and turnover mt-RNAs. Mature mt-mRNAs engage mitochondrial ribosomes and use translation factors, mt-tRNAs and tRNA synthetases to support translation initiation, elongation, termination and ribosome recycling to ensure translation proceeds efficiently and accurately. The mitochondrial ribosomes are anchored to the inner membrane and associate with OXA1L and cardiolipin to facilitate efficient, co-translational insertion of hydrophobic mitochondrial OXPHOS proteins into the inner membrane. Image made using BioRender.com.

Nuclear encoded post-translationally imported RNA-binding proteins control the transcription, processing, stability and turnover of mitochondrial transcripts to ensure their translation on mitoribosomes [1] (Fig. 1). The mitochondrial RNA polymerase (POLRMT) supported by the mitochondrial transcription elongation factor (TEFM) promote the transcription of the mitochondrial DNA (mtDNA), generating genome length polycistronic transcripts [42–44]. These are essential proteins and recently pathogenic mutations in *POLRMT* and *TEFM* that cause mitochondrial diseases have validated their roles in transcription elongation further [45, 46]. Heart- and skeletal muscle-specific *Polrmt* and *Tefm* deletion in mice leads to profound cardiomyopathy and early onset death caused by loss of mitochondrial transcription and thereby lack of mitochondrial translation and OXPHOS assembly [42, 44]. Impaired OXPHOS function as a consequence of reduced transcription was also found in patients with pathogenic mutations in *POLRMT* and *TEFM* that resulted in early onset neurological and muscle disorders as well as premature death in some cases [45, 46]. Although full body deletions of these genes cause embryonic lethality [42, 44], knocking in the identified *POLRMT* and *TEFM* pathogenic mutations in mice could provide valuable models to assess the tissue-specific effects and disease progression.

In mammals, mitochondrial tRNAs (mt-tRNA) act as “punctuation marks” spanning the mRNAs and rRNAs on the mitochondrial polycistronic transcripts [47]. The RNase P complex (composed of MRPP1/TRMT10C, MRPP2/HSD17B10 and MRPP3/PRORP) cleaves the 5′ ends of tRNAs [48, 49] and the RNase Z, ELAC2, cleaves the 3′ ends [50], thereby releasing the individual mt-mRNAs, mt-tRNAs and mt-rRNAs. Pathogenic mutations that cause mitochondrial disease have been identified in all of the mtRNA processing components [51–55], and mouse deletions of *Mrpp2, Mrpp3* and *Elac2* have been generated [49, 50, 56]. Like POLRMT and TEFM, MRPP3/PRORP and ELAC2 are essential proteins with non-redundant functions that cause very early cardiomyopathy and premature death in mice that model that seen in the patient mutations. Mutations in the MRPP2/HSD17B10 protein cause progressive neurodegeneration, retinopathy and cardiomyopathy [55]. The MRPP2 protein performs dual functions, both in catalyzing the second dehydrogenation reaction in the beta-oxidation cycle of 2-methylbutyryl-CoA in isoleucine metabolism [57] and as a component of the RNase P complex [48]. Constitutive knockout of MRPP2 is embryonic lethal in mice, whereas tissue-specific MRPP2 knockout in noradrenergic neurons and endothelial cells caused mitochondrial dysfunction and apoptosis [56]. The MRPP3 and ELAC2 models both showed that RNA processing is linked to mitochondrial translation via mitoribosome assembly, and in the absence of RNA processing, protein synthesis and OXPHOS function are diminished. Interestingly, a common genetic variant in the *MRPP3* gene [58], was functionally validated as a predisposing factor for high fat-diet induced insulin resistance, due to a moonlighting role of the RNase P complex with the LETM1 cation transporter [59]. Another common genetic variant identified in *ELAC2* as the second most common susceptibility factor for prostate cancer [60] was introduced recently in mice [61]. The Ala541Thr variant led to reduced ELAC2 activity that resulted in impaired mitochondrial and nuclear tRNA processing and validated the role of ELAC2 [50], causing prostate hyperplasia and inflammation with age, but not prostate cancer [61]. Prostate-specific deletion of *Elac2* resulted in similar defects and prostate inflammation. Prostate cancer developed in both models when the mutations were bred on the transgenic adenocarcinoma of mouse prostate (TRAMP) background, implicating *ELAC2* mutations as susceptibility factors for prostate cancer in the presence of additional genetic insults. Genetic variants in key mitochondrial RNA-binding proteins along with pathogenic patient mutations reveal the importance of mitochondrial gene expression in diverse diseases and tissues and provide valuable models of common metabolic disorders and cancer.

**Figure 2:**
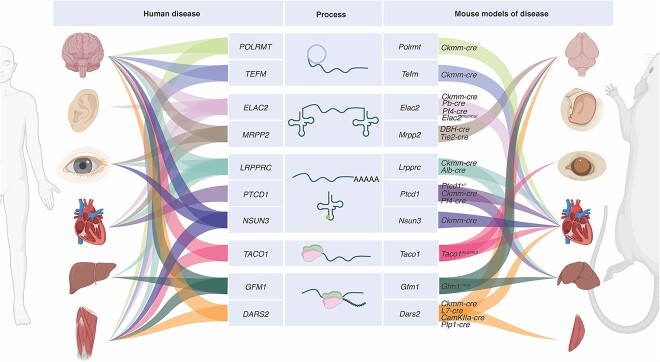
Mouse models of diseases that result in impaired mitochondrial translation. Diseases associated with mitochondrial translation defects cause multi-systemic disorders, with diverse phenotypes and varied severity. Different mouse models have been developed to understand disease pathologies that are a consequence of impaired mitochondrial gene expression and translation, by knocking in equivalent or orthologous pathogenic mutations identified in patients or by generating tissue-specific deletions of the disease genes. Constitutive knockout of translation factors with non-redundant function in mice generally results in embryonic lethality, therefore Cre-*loxP* recombination under the control of transcription factor drivers has been used to generate tissue-specific deletions of translation factors. Summarized are the effects of human disease gene mutations and corresponding mouse models developed to understand their pathology. Image made using BioRender.com.

The suite of enzymes that modify and stabilize mitochondrial transcripts can also affect the rate of protein synthesis and many of them have been implicated in diseases including mitochondrial diseases [62]. However, the loss or mutations in only a few of these genes have been studied in mouse models (Fig. 2). PTCD1 was identified as an essential factor required for 16S rRNA stability and early ribosome assembly [63, 64] and mutations in *PTCD1* have been linked to cardiomyopathy in patients [65]. Cardiomyopathy is also a major pathogenic feature of heterozygous *Ptcd1* knockout mice and is the cause of premature death in tissue-specific homozygous *Ptcd1* knockout mice [63, 64]. Recently, tissue-specific loss of both *Elac2* and *Ptcd1* in mouse megakaryocytes and platelets revealed that mitochondrial RNA metabolism is required for platelet activation and thrombus formation and their loss caused thrombocytopenia, increased bleeding time and higher platelet turnover [66]. It is interesting to note that in platelets, which are devoid of a nucleus, the existing RNA-binding protein complement can regulate mitochondrial gene expression, which is required for platelet activation.

The best known and most studied mitochondrial RNA-binding protein, LRPPRC, when stabilized in a complex with SLIRP [67, 68], acts as an RNA chaperone, to stabilize mt-mRNAs [67–70]. Specifically, the binding of the LRPPRC-SLIRP complex relaxes mRNA secondary structures to expose the required sites for polyadenylation, stabilization and translation [50]. Within a cell and between tissues types, mt-mRNA transcripts are maintained at varied abundances [69, 71] and the LRPPRC/SLIRP complex displays different affinity for the polyadenylated mt-mRNAs [70]. Mutations in LRPPRC have been linked to Leigh Syndrome, French Canadian Type (LSFC) [72, 73] and cause Complex IV dysfunction that most severely affects brain and liver OXPHOS [74, 75]. Constitutive homozygous *Lrpprc* deletion in mice is embryonic lethal [70], which validates its essential role for mt-RNA stability. Liver-specific *Lrpprc* knockout mice have growth delay, and severe mitochondrial hepatopathy [76], whereas heart- and skeletal muscle-specific loss of LRPPRC leads to early onset cardiomyopathy and premature death [70], providing useful models for the pathogenesis of the disorder in these tissues. Interestingly, in the cardiac-specific *Lrpprc* knockout mouse model, Complex V dysfunction drives the disorder rather than Complex IV [70, 77], which is not observed in patients [75] and appears to be a mouse specific response [76]. In the liver-specific *Lrpprc* knockout mice, assembly of both Complex IV and V is disrupted, however, OXPHOS capacity and liver function are preserved despite a global mitochondrial translation defect and a significant Complex IV assembly defect. Mechanisms operating to maintain liver function include upregulation of mitochondrial and mitoribosome biogenesis and stress response pathways, that appears to share a common feature with the error-prone MRPS12 mice [25]. Additionally, the residual Complex IV is stabilized in supramolecular respirasomes, also identified in patient cells, likely through remodeling of mitochondrial glycerophospholipids that are required for complex assembly and stability in the inner membrane [78]. Pathogenic variants in the *TACO1* gene that encodes a translational activator of cytochrome c oxidase 1 (*MT-CO1*) mRNA, led to late-onset Leigh syndrome, also caused by a specific Complex IV deficiency [79, 80]. When modeled in mice, a *Taco1* mutation that resulted in TACO1 loss caused similar late-onset pathologies affecting vision, motor function and learning capacity [81] that could be further exacerbated by infection mimicking sudden triggers of mitochondrial diseases [82]. TACO1 was shown to bind the *MT-CO1* mRNA to facilitate its association with the ribosome and its loss caused an isolated Complex IV deficiency, consistent with the role of COXI in the initial assembly of Complex IV [81]. The particularly severe involvement of Complex IV described in LSFC caused by *LRPPRC* mutations, is difficult to explain by a simple global translation defect, given Complex I contains seven mitochondrial translated subunits, while Complex IV has only has three. The intricacies of respiratory complex assembly, including the association of complexes into higher order assemblies, including super-complexes and mega-complexes [83], likely contributes to this complexity. Within these assemblies, complexes are not incorporated in equal proportions, suggesting that certain complexes are more abundant in the inner membrane than others [84]. Additionally, the half-lives of the protein subunits vary substantially [85]. These factors regulating respiratory complex subunit abundance likely lead to the differential complex susceptibility to translation defects, and likely contribute to the differential impacts on respiratory complex assembly identified in each disorder.

The translation factors TFB1M, NSUN4, MTERF4, DARS2 and MTERF3 involved in rRNA modification and stability, mitoribosome assembly and fidelity of translation have been deleted specifically in heart and skeletal muscle, presenting with cardiomyopathies and premature death [34, 86–89]. These models have helped reveal the molecular and physiological roles for these proteins and recent studies have focused on identifying the tissue-specific defects found in disease. Unlike most mutations in mitochondrial translation proteins that cause predominantly neuronal defects, mutations in DARS2, encoding the mitochondrial aspartyl-tRNA synthetase, cause white matter disease-leukoencephalopathy with brainstem and spinal cord involvement, and lactate elevation (LBSL) characterized by progressive spastic ataxia and multiple long-tract involvements. Therefore, DARS2 was specifically depleted in forebrain-hippocampal neurons or myelin-producing cells; these mice showed that adult neurons are depleted as a result of severe mitochondrial dysfunction and model neurological disorders found in patients, where myelin-producing cells were resistant to cell death despite having an OXPHOS defect [90, 91]. Further analyses of the specific cell-type involved in the pathology stimulated the production of a conditional Purkinje cell-specific *Dars2* deletion that resulted in early and profound loss of Purkinje cells, causing deteriorating motor skills in the mice [92]. In a mouse model developed from the mtDNA mutator mice (homozygous knock-in mice that express a proof-reading-deficient version of the nucleus-encoded catalytic subunit of mtDNA polymerase (*PolgA*) [93], mice with a mutation in tRNA^ALA^ were studied and shown to develop cardiomyopathy as a result of disrupted translation [94]. Interestingly, this study identified that highly proliferative tissues have the capacity to select against high levels of the *tRNA^ALA^* mutation, while less proliferative tissues maintain a constant level of mutation [94]. Furthermore, a recent study has demonstrated that cell lineage-specific mitochondrial gene expression emerges early in development, and linage specific transcriptional responses determine how tissues respond to translation dysfunction [95]. These models highlight the importance of tissue- and cell-specific analyses of gene mutations to reveal their physiological and pathological roles and establish specific target tissues for therapeutic intervention.

## Modeling the role of mitochondrial translation factors in disease

Translation factors control the progress and fidelity of translation of mt-mRNAs through four main stages: initiation, elongation, termination and ribosome recycling. Many models have been developed to study these factors, and the functional consequences of mutations in the genes that code them.

### Translation initiation factors

Translation initiation factors assist with the recruitment of mature mt-mRNAs to the 28S subunit, and positioning of the start codon in the peptidyl decoding site, paired to the anticodon of the initiator tRNA [96]. While the bacterial system has three initiation factors, IF1, IF2 and IF3, the formation of the mitochondrial translation initiation complex requires only MTIF2 and MTIF3 [16, 97]. MTIF2 enhances initiator tRNA binding to the small subunit in the presence of a start codon [16, 98, 99], and functionally replaces IF1, preventing premature association of elongator tRNAs in the A-site during initiation [100]. Deletion of the *Mtif3* gene in mice caused embryonic lethality whereas heart- and skeletal muscle-specific loss of MTIF3 causes mitochondrial dysfunction that leads to cardiomyopathy [2] or platelet-specific loss that causes thrombocytopenia and increased bleeding [66], indicating that mitochondrial protein synthesis is required in diverse tissue and cell types. MTIF3 regulates the rate of translation initiation, and is required for correct mRNA positioning in the preinitiation complex, and the removal of prematurely bound initiator tRNA [2, 101, 102]. These novel functionalities are likely facilitated through its evolved N- and C-terminal extensions [103, 104]. While the bacterial system relies on interaction of the upstream Shine-Dalgarno sequence with the complementary region of the 16S rRNA for start codon recognition [105], mt-mRNAs have only a few nucleotides or no 5′-UTRs [106, 107], and the mammalian mitoribosome has evolved to recognize leaderless mRNAs [108]. Finally, both canonical and non-canonical start codons must be recognized by the preinitiation complex, likely facilitated by the mitochondria-specific ribosomal proteins and partly through tRNA modifications, which assist with deciphering non-canonical codons. In mitochondria, the 22 tRNAs possess 18 types of modifications [109]. It has been demonstrated through mouse knockout models, that deletion of those enzymes that modify tRNAs at the first nucleotide of the anticodon are particularly severe [110–112]. The recognition of the canonical AUG, and non-canonical AUA methionine codons is facilitated through the 5-formylcytidine (f^5^C) modification on the first nucleotide of the tRNA-Met anticodon by NSUN3 and ALKBH1 [113–115]. Mutations in NSUN3 have been described in patients with muscle weakness, opthalmoplegia, lactic acidosis and developmental delay [116, 117]. A heart-specific *Nsun3* deletion in mice caused heart defects that became exacerbated with age [111], which matched with reports of age-related heart disease in a patient with tRNA-Met mutation [118], which decreased the efficiency of NSUN3-mediated methylation to 40% [115].

### Translation elongation factors

Three proteins: mitochondrial elongation factor G (GFM1), mitochondrial translation elongation factor Tu (TUFM) and mitochondrial translation elongation factor Ts (TSFM), control the progression of mitochondrial translation elongation [97]. GFM1 evolved from bacterial EF-G, which performs two distinct functions, at elongation and ribosome recycling [119]. In mitochondria two EF-G homologs now carry out these functions independently; one of those is GFM1 which regulates elongation, while GFM2 promotes translation termination and ribosome recycling [120, 121]. GFM1 has evolved N- and C-terminal extensions that facilitate its interaction with the mitochondrial 16S rRNA to control tRNA translocation [122]. The TUFM protein is a highly conserved GTPase that forms a ternary complex with GTP and aminoacylated-tRNA, to promote binding at the A-site [123]. Following tRNA delivery, TSFM complexes with TUFM to facilitate its release from the ribosome [124].

Mutations in the *GFM1* gene cause diseases with a primary Leigh-like presentation, some patients die in infancy, while in other cases the disease remains stable [125–127]. Mutations in the central region of the protein have been associated with hepatic failure, while mutations at the periphery have been associated with encephalopathy [128]. A pathogenic variant in TUFM, located in the domain responsible for TUFM-TSFM interaction, caused lactic acidosis and dilated cardiomyopathy without encephalopathy [129]. Interestingly, a patient with a mutation in the corresponding domain of TSFM, presented with infantile encephalocardiomyopathy and sensorineural hearing loss [130], thereby indicating that even disruption to the same interaction site can lead to distinct disease presentations, consistent with the heterogeneity typically observed in mitochondrial diseases. To explore these complex human disease presentations, a pathogenic variant, R671C, was introduced into the *Gfm1* gene in mice, and found to cause a significantly milder disease than that observed in patients [131]. The reason for this was unclear, because residue R671 in both the human and mouse GFM1 protein is essential for its stability and weakens its interaction with the ribosome. While patients developed hepatoencephalopathy, the mutant mice displayed only a mild molecular phenotype in the liver, including significant decrease in Complex IV activity and a decreased translation rate [131]. In patients with fatal hepatopathy due to a *GFM1* mutation, OXPHOS dysfunction appeared to correlate with residual protein abundance, which was absent in the liver, but maintained at 60% of control in heart, therefore maintaining sufficient translation capacity in this tissue [132]. This difference in protein stability was not apparent in the mouse model, because GFM1 abundance was reduced by approximately 85% in heart, liver, kidney and brain, but still only caused dysfunction of liver mitochondria [131]. To produce a more comparable model to the human disease, compound heterozygous mice were generated, by knocking out one *Gfm1* allele, and inserting the R671C mutation into the other, which provided the first *in vivo* model of hepatoencephalopathy [131]. In this model, Complex IV remained most significantly affected, in both liver and brain, with an additional moderate effect on Complex I. In the heart, despite strong reduction of GFM1, there was only a mild effect on Complex IV, matching the patient phenotype, suggesting that heart function was somehow protected. Upregulation of other elongation factors was observed as an adaptive response operating in patient hearts to protect this tissue from dysfunction [132], but there was no evidence of this mechanism in mice [131].

An additional factor potentially modulating tissue specific disease is the varied abundance of elongation factors between tissues, which could influence the ability of each to buffer translation defects. The ratio of TUFM to TSFM expression in the muscle, liver and fibroblasts is similar, whereas the ratio of these factors is 1:6 in the heart [132]. In a case report of a patient with a mutation in the *TUFM* gene, they presented with a tissue-specific disease presentation impacting the heart, which may be particularly susceptible to TUFM depletion [129]. Interestingly, in other cases of *TUFM* mutations, encephalopathy was the primary phenotype [123, 133], and so investigation of the expression ratio of elongation factors in the brain would be valuable, to examine if the expression pattern is similar to that of the heart. The tissue-specific effects on mitochondrial translation are particularly interesting and clearly play a significant role in the pathology of these disorders, however, the mechanisms governing these differences remain to be unraveled.

### Translation termination and ribosome recycling factors

When a stop codon enters the A-site in the mitoribosome it signals the end of the open reading frame, and stimulates recruitment of release factors to catalyze hydrolysis of the ester bond between the nascent chain and P-site tRNA [97]. While the bacterial system uses two release factors, RF1 and RF2 [134], four release factors have been identified in the human mitochondrial translation system, MTRF1, MTRF1L, ICT1 and MTRFR [135–143]. Despite the near universal conservation of the standard genetic code, the mitochondrial genetic code has diverged and two non-canonical stop codons, AGA and AGG, specify the end of the open reading frames of the *MT-CO1* and *MT-ND6* mRNAs. Structural analysis of the termination complex revealed that MTRF1L exclusively binds the canonical UAA and UAG stop codons [97, 144], while ICT1 can bind specifically to ribosomes with an empty A site that resembles a no-stop scenario, in which the ribosome is stalled at the 3′ end of a truncated mRNA [97]. MTRFR is a rescue factor that works with MTRES1 (previously C6ORF203) to release the nascent chain and tRNA from stalled ribosomes [137]. Non-canonical stop codons are recognized by MTRF1, which releases MT-CO1 [145–147] and MT-ND6 [145, 147] from the ribosome, and is capable of recognizing their con-canonical stop codons via its N-terminal extension [147]. To complete the process, mitochondria require two further factors; MRRF and GFM2, to promote ribosome recycling [97, 121, 148], preparing the subunits for another round of translation and completing the translation cycle.

In a case report of a patient with compound heterozygous mutation of *MTRFR*, they presented with spastic paraplegia, decreased vision and optic atrophy [149]. Knockdown of MTRFR and ICT1 in Hela cells showed that both are required for cell viability and mitochondrial function, however, there were distinct consequences of each knockdown, indicating that they act differently in rescuing stalled ribosomes [150]. Fibroblasts from patients with *MTRFR* mutation, show a global and uniform decrease in mitochondrial translation, which caused decreased OXPHOS complex assembly [136]. Mutations in recycling factors have also been linked to mitochondrial disease. In a study characterizing biomarkers of early-stage Parkinson’s disease, MRRF was found to be significantly enriched in patients compared to controls [151]. *GFM2* variants have been associated mainly with Leigh syndrome type disease [152], as well as muscular weakness, developmental delay and brain abnormalities [153]. The severity of these OXPHOS defects appeared to correlate with location of the mutation in conserved protein regions [153]. Furthermore, mutations in *GFM2* do not cause a decrease in *de novo* mitochondrial translation, because of their role following translation completion, which demonstrates that disruption to the release of peptides from the ribosome is also a mechanism causing mitochondrial OXPHOS dysfunction. These diseases observed in patients are severe, and commonly affect the central nervous system. Further investigation of termination and recycling factors using *in vivo* models will be valuable and help to explain the mechanisms leading to disease.

## Conclusions

Although almost all of the approximately 1100–1400 different mitochondrial proteins that make up mammalian mitochondria are encoded by the nuclear genome, the importance of the 13 mitochondrially-encoded proteins is highlighted by the observation that their translation requires ~20% of the mitochondrial proteome [154]. Key proteins involved in mitochondrial translation have been identified through their homologies to ancestral bacterial proteins, via biochemical purifications, and as disease causing mutations in mitochondrial disease patients, however, linking these component parts to their functional and physiological roles has been pioneered through the use of mouse models [155]. Before the discovery of CRISPR gene editing, mouse models provided the only practical approach to generate complete gene knockouts, and unveiled the functions of many key components of the mitochondrial gene expression pathway [2, 25, 34, 42, 44, 49, 50, 56, 63, 64, 70, 81, 86–89, 131] (Table 1). Beyond this, mouse models with altered mitochondrial gene regulation have proven to be valuable tools to understand underlying disease pathologies. In this regard mice have several key advantages, most critically that they are genetically and physiologically very similar to humans—with the vast majority of genes having exact one-to-one orthologs [156] and the same organ systems conserved in both organisms. Their body size enables mice to be housed in small and homogeneous spaces, so that their environments can be precisely controlled. Furthermore, there are well-established testing systems for physiological and behavioral tests have been devised to match those examining important human traits.

**Table 1:** Mouse models of translation defects caused by loss or mutation of proteins that regulate mitochondrial gene expression.

Mouse Model	Molecular insights	Mouse phenotype	Ref
Transcription			
**POLRMT**			
***β*** *-actin-cre* homozygous KO (ubiquitous)		Embryonic lethal.	[44]
*Ckmm-cre* homozygous KO (cardiac)	Regulates mt-DNA replication, controls switch between primer formation for mt-DNA replication and gene expression.	Dilated cardiomyopathy. Death by 6 weeks.	[44]
**TEFM**			
***β*** *-actin-cre* homozygous KO		Embryonic lethal.	[42]
*Ckmm-cre* homozygous KO	A general transcription elongation factor, necessary for both gene transcription and replication primer formation.	Cardiomyopathy. Death by 9 weeks.	[42]
**TFAM**			
***β*** *-actin-cre* homozygous KO	Regulates transcription and replication of mt-DNA.	Embryonic lethal. Embryos lack mt-DNA and cardiac, neural, optic and somite development are affected.	[159]
***β*** *-actin-cre* heterozygous KO	Modulates mt-DNA copy number and expression, supports conclusions of earlier work *in vitro.*	Heart more affected than kidney, liver or muscle. Reduced mt-DNA copy number. Respiratory chain malfunction.	[159]
*Mlc1f*-*cre* homozygous ko (myosin light chain *1f* locus)	A substantial increase in mitochondrial mass in skeletal muscle can partly compensate for reduced respiratory chain function by maintaining overall ATP production.	Mitochondrial myopathy with ragged-red fibers, accumulation of abnormal mitochondria, progressively deteriorating respiratory chain function, and reduced muscle-force production. Mice need to be culled at 20 weeks.	[160]
*Ckmm-cre* homozygous KO	Reproduces important pathophysiological features of mitochondrial cardiomyopathy.	Dilated cardiomyopathy and atrioventricular heart conduction blocks. Death by 2–4 weeks.	[161]
*Myhca-cre* homozygous KO (α-myosin heavy chain)	Highlights the importance of modifying genes in determining knockout lifespan.	Disease onset during embryogenesis. Dilated cardiomyopathy with atrioventricular heart conduction blocks. 75% neonatal death, 25% delayed death.	[162]
*CAMKIIα-cre* homozygous KO (Neuronal)	Model of late onset neurodegeneration and the role for respiratory chain deficiency in neurodegeneration and aging.	Late onset, rapidly progressive neurodegeneration and hippocampal/neocortex cell death at 20 weeks.	[163]
*CD4-cre* homozygous KO (Adaptive immune system)	Describes mechanism by which mitochondria regulate lysosome function to preserve T cell differentiation and effector functions.	Decreased cellular mtDNA content alters mitochondrial metabolism and is associated with impaired endolysosomal function, abnormal accumulation of sphingomyelins and increased pro-inflammatory T cell responses.	[164]
*Yfp-cre* Homozygous KO (Regulatory T cells)	Required for TFAM-mediated mitochondrial respiration in T-cells to regulate inflammation and anti-tumor immunity.	*Tfam* deletion in T-cells affects T-cell homing and stability, resulting in tissue inflammation in colitis, but enhances tumor rejection.	[165]
*adiponectin-Cre* homozygous ko (Adipocyte)	Isolated mitochondrial dysfunction in adipose tissue can lead to lipodystrophy with metabolic syndrome and cardiovascular complications.	Mice are resistant to weight gain, develop insulin resistance, hypertension and cardiac hypertrophy.	[166]
*aP2-Cre* homozygous KO (adipose)	TFAM deletion in adipose tissue increases mitochondrial oxidation, with positive metabolic effects, suggesting regulation of adipose tissue mitochondria may be a therapeutic target for obesity treatment.	Mice exhibit higher energy expenditure and are protected from age- and diet-induced obesity, insulin resistance, and hepatosteatosis, despite a greater food intake.	[167]
*RIP-cre* homozygous KO (rat insulin-2 promoter) (pancreatic β-cells)	Model for β-cell pathology of human mitochondrial diabetes. Also provides genetic evidence for a critical role of the respiratory chain in insulin secretion.	Diabetes onset from 5 weeks and severe mt-DNA depletion, deficient oxidative phosphorylation and abnormal mitochondria in islet cells.	[168]
Overexpression of human TFAM in mice	Expression of human TFAM in mice increased the amount of mtDNA almost in parallel with the increase in the TFAM. Ameliorates the typical symptoms of mitochondrial disease, by increasing mtDNA copy number.	Improves lifespan and decreases disease severity.	[169–172]
Overexpression of mouse TFAM in mice	TFAM acts as a general gene repressor of mtDNA expression and this effect can be counterbalanced by tissue-specific expression of regulatory factors. Modulation of TFAM levels serves as a global mechanism to regulate mitochondrial gene expression likely by influencing nucleoid compaction.	Moderately increased TFAM and mtDNA levels are well tolerated *in vivo.* Strong TFAM overexpression results in postnatal lethality by 5 weeks.	[173, 174]
**MTERF1**			
***β*** *-actin-cre* homozygous KO (*Mterf1a* and Mterf1b)	Prevents L-strand transcripts from proceeding around the mtDNA circle and causing transcription interference at the LSP promoter from which they originated.	Mice were healthy, loss of MTERF1 had no impact on oxidative phosphorylation capacity.	[175]
RNA processing			
**ELAC2**			
***β*** *-actin-cre* homozygous KO		Embryonic lethal.	[50]
*Ckmm-cre* homozygous KO	Facilitates 3′ mt-tRNA cleavage and balanced maintenance of C/D box snoRNAs, miRNAs, and tRNA fragments. Also links RNA processing to translation through mitoribosome assembly	Dilated cardiomyopathy. Death by 4 weeks.	[50]
*Pf4-cre* homozygous KO (megakaryocytes and platelets)	Mitochondrial RNA metabolism is required for platelet activation and thrombus formation.	Thrombocytopenia, increased bleeding time and higher platelet turnover.	[66]
*Pb-cre* homozygous KO (prostate)	Models prostate-specific mitochondrial dysfunction.	Enlargement and inflammation of the prostate and nodule formation. Normal lifespan.	[61]
Homozygous A537T (equivalent to human prostate cancer susceptibility variant)	Models ELAC2 mutation from human patients.	Enlargement and inflammation of the prostate and nodule formation. Normal lifespan. If combined with a secondary genetic insult the onset and progression of prostate cancer are exacerbated.	[61]
**MRPP2/HSD17B10**			
*Tie2-cre* homozygous KO (Endothelial)	HSD10 is essential for structural and functional integrity of mitochondria, independently of its enzymatic activity.	Spleen and vasculature dysfunction. Death by 25 weeks.	[56]
*DBH-cre* homozygous KO (Noradrenergic)	HSD10 has a protective effect on mitochondrial integrity.	Disruption to mitochondrial structural integrity in the CNS and the PNS. Death by 26 weeks.	[56]
**MRPP3/PRORP**			
***β*** *-actin-cre* homozygous KO		Embryonic lethal.	[49]
*Ckmm-cre* homozygous KO	Facilitates 5′ mt-tRNA cleavage and links RNA processing to translation through mitoribosome assembly.	Cardiomyopathy. Death by 11 weeks	[49]
RNA stability			
**TFB1M**			
***β*** *-actin-cre* homozygous KO		Embryonic lethal.	[87]
*Ckmm-cre* homozygous KO	Dimethyltransferase that modifies 12S rRNA.	Cardiomyopathy. Death by 24 weeks.	[87]
***β*** *-actin-cre* heterozygous KO	TFB1M is a nonredundant dimethyltransferase. Adenine dimethylation plays a critical role in ribosome maintenance.	Reduced insulin secretion in response to glucose.	[176]
TFB1M overexpression	Overexpression of the mitochondrial methyltransferase TFB1M in mice does not impact mitoribosomal methylation status or hearing,	No changes were detected in metabolism, cardiovascular function, lung function, eye function, grip strength or rotarod performance.	[177]
**PTCD1**			
***β*** *-actin-cre* homozygous KO		Embryonic lethal.	[63]
***β*** *-actin-cre* heterozygous KO	Haploinsufficiency causes increased RNA metabolism, in response to decreased protein synthesis and impaired RNA processing that affect the biogenesis of the respiratory chain.	Age-induced obesity, liver steatosis and hypertrophic cardiomyopathy.	[63]
*Ckmm-cre* homozygous KO	16S rRNA binding protein required for its stability, pseudouridylation and correct biogenesis of LSU.	Dilated cardiomyopathy and mild muscle myopathy. Death by 10 weeks	[64]
*Pf4-cre* homozygous KO	Mitochondrial RNA metabolism is required for platelet activation and thrombus formation.	Thrombocytopenia, increased bleeding time and higher platelet turnover.	[66]
**LRPPRC**			
Homozygous KO		Embryonic lethal.	[70]
Homozygous KO rescued with *BAC-LRPPRC–Flag*	The homozygous *Lrpprc* knockout can be rescued by the BAC transgene encoding LRPPRC with a C-terminal Flag tag, showing that the knockout of the *Lrpprc* gene is causing the observed embryonic lethality.	Mice are viable.	[70]
*Ckmm-cre* homozygous KO	LRPPRC forms an RNA-dependent complex with SLIRP, to stabilize a pool of non-translated mitochondrial mRNAs. Loss of LRPPRC causes aberrant mitochondrial translation with excessive translation of some transcripts and no translation of others.	Cardiomyopathy. Death by 16 weeks.	[70, 77]
*Alb-cre* homozygous KO (Hepatocytes)	LRPPRC deficiency caused destabilization of polyadenylated mRNAs, altered mitochondrial ultrastructure, and a severe CIV and CV assembly defect, impairment of long-chain fatty acid oxidation, dysregulation of the mitochondrial PTP, and alteration of trans-membrane H2O2 diffusion due to CV defect and altered membrane lipid composition.	Generalized growth delay, no signs of overt liver failure and capacity of the ETC is preserved, despite global mitochondrial translation defect.	[76]
*Alb-cre* homozygous KO (Hepatocarcinogen treated)	LRPPRC suppresses genome instability and hepatocellular carcinomas and promotes survivals in mice by sustaining Yap-P27-mediated cell ploidy and P62-HDAC6-controlled autophagy maturation.	LRPPRC depletion synergistically enhances diethylnitrosamine-induced DNA damage, genome instability, and further tumorigenesis so that LRPPRC knockout mice develop more and larger hepatocellular carcinomas and have a shorter lifespan.	[178]
Heterozygous KO	LRPPRC does not directly regulate mtDNA transcription but rather acts as a post-transcriptional regulator of mammalian mtDNA expression.	Viable, fertile and healthy.	[179]
LRPPRC overexpression	Mice with moderately altered expression of LRPPRC, corresponding to a predicted normal physiological range are healthy with no obvious phenotypes.	Viable, fertile and healthy.	[179]
**SLIRP**			
***β*** *-actin-cre* homozygous KO	Regulates the rate of translation and protects LRPPRC from degradation.	Viable and healthy with only mild weight loss. Male mice displayed decreased fertility.	[67, 180]
**NSUN3**			
***β*** *-actin-cre* homozygous KO	Mt-tRNA anticodon modification is essential for mammalian embryonic development.	Embryonic lethal. Alive at E10.5, die before E12.5.	[111]
*Myh6-Cre* homozygous KO (Myosin heavy chain promoter)	Tissue-specific loss of a single mitochondrial tRNA modification can induce tissue aberration that worsens in later adulthood.	Mild heart abnormalities that become more apparent with age.	[111]
**MTO1**			
*Alb-cre* homozygous KO (Hepatocytes)	MTO1 mediated taurine modification of mt-tRNAs is indispensable for protein translation.	Embryonic lethal.	[110]
*Myh6-Cre* homozygous KO (Myosin heavy chain promoter)		Embryonic lethal.	[110]
**MTU1**			
CAG-cre Homozygous KO		Embryonic lethal by E7.5.	[112]
*Alb-cre* homozygous KO (Hepatocytes)	MTU1 dependent 2-thiolation in mt-tRNAs is indispensable for translation and MTU1 deficiency is a primary cause of RILF.	Mice are viable. Significant liver injury is present in these mice.	[112]
tRNA charging			
**DARS2**			
***β*** *-actin-cre* homozygous KO		Embryonic lethal.	[34]
*Ckmm-cre* homozygous KO	Results show that mitochondrial dysfunction is sensed independently of respiratory chain deficiency, questioning the current view on the role of stress responses in mitochondrial diseases.	Cardiomyopathy and skeletal muscle atrophy Premature death by 7 weeks.	[34]
*Plp1-CreERT* homozygous KO (Oligodendrocytes)	Glial cells are not the main target of DARS2 deficiency.	No neurodegeneration or neuroinflammation.	[90]
*CAMKIIα-cre* homozygous KO	Disrupted mitochondrial protein synthesis triggers neuroinflammation.	Apoptotic cell death and brain atrophy. Death by 32 weeks.	[91]
*L7-cre* homozygous KO (Purkinje)	DARS2 is indispensable for Purkinje cell survival and protects against cerebellar ataxia.	Severe loss of Purkinje cells by 15 weeks and rapidly deteriorating motor skills.	[92]
**HARS2**			
***β*** *-actin-cre* homozygous KO		Embryonic lethal	[181]
*Gfi1-cre* homozygous KO (Cochlear hair cells)	Models hearing loss seen in patients with Perrault Syndrome.	Progressive hearing loss, becoming deaf by 60 days.	[181]
**FARS2**			
Homozygous KO	*FARS2* function is required for embryonic neurogenesis.	Embryonic lethal. Can develop definitive endodermal and mesodermal layers, but not the ectoderm.	[182]
Homozygous D142Y (loss of function mutation)	*Fars2* function is required for embryonic neurogenesis. D142Y produces a KO-like phenotype in mice.	Embryonic lethal. Can develop definitive endodermal and mesodermal layers, but not the ectoderm.	[182]
Heterozygous D142Y		Viable.	[182]
*Nestin-cre* homozygous KO (Neuronal)	Impaired mitochondrial function due to *Fars2* deficiency affects neuronal development and potentiates neuronal cell apoptosis.	Brain morphology shows enlarged ventricle and reduced cortical thickness. Death shortly after birth.	[182]
**AARS2**			
Homozygous C744A	Editing function of mtAlaRS is an essential protein quality control mechanism required for mouse development.	Embryonic lethal. Mild editing deficiency.	[183]
Heterozygous C744A		Healthy.	[183]
Homozygous V755E		Embryonic lethal. Severe editing deficiency.	[183]
Heterozygous V755E		Healthy.	[183]
**WARS2**			
Homozygous KO		Embryonic lethal.	[184]
Homozygous V117L (loss of function mutation)	Translation inhibition causes heart-specific ISR activation, increasing FGF21 levels and causing systemic changes in metabolism.	Hearing loss, reduced adiposity, adipose tissue dysfunction, and hypertrophic cardiomyopathy.	[184]
Mitoribosome			
**MRPS5**			
Homozygous V338Y	MRPS5 function regulates translation accuracy.	Noise induced hearing damage and anxiety related behavioral changes.	[24]
**MRPS12**			
Homozygous K72I	MRPS12 function regulates translation accuracy.	Error prone translation.	[25]
Homozygous K71T	MRPS12 function regulates translation accuracy.	Hyper accurate translation. Cardiomyopathy.	[25]
**MRPS34**			
Homozygous L68P	Required for the stability of the 12S rRNA, the small ribosomal subunit and actively translating ribosomes.	Age induced heart and liver dysfunction.	[185]
**MTERF4**			
***β*** *-actin-cre* homozygous KO		Embryonic lethal.	[86]
*Ckmm-cre* homozygous KO	Controls mitoribosome biogenesis and translation. Forms a complex with NSUN4 to facilitate its recruitment to the LSU.	Cardiomyopathy. Death by 21 weeks.	[86]
**NSUN4**			
***β*** *-actin-cre* homozygous KO		Embryonic lethal. Retarded growth, no clearly discernible anatomical structures at E8.5.	[88]
*Ckmm-cre* homozygous KO	NSUN4 alone can methylate C911 12S rRNA, while it must interact with MTERF4 to facilitate monosome assembly.	Cardiomyopathy. Death by 25 weeks.	[88]
**CLPP**			
***β*** *-actin-cre* homozygous KO	Essential role in determining the rate of mitochondrial protein synthesis by regulating the level of mitoribosome assembly. Without CLPP, ERAL1, a putative 12S rRNA chaperone, strongly associates with the SSU preventing monosome formation.	Knockout mice were not born in Mendelian proportions, indicating essentiality in some critical period during development, and postnatal tolerance to CLPP deficiency. Mice develop Perrault syndrome, but have a normal lifespan.	[186, 187]
**ERAL1**			
Homozygous KO		Embryonic lethality.	[188]
Heterozygous KO	ERAL1 positively regulates RNA virus-triggered innate immunity.	Healthy, normal lifespan. More susceptible to VSV infection.	[188]
**MTERF3**			
***β*** *-actin-cre* homozygous KO		Embryonic lethal.	[89]
*Ckmm-cre* homozygous KO	Biogenesis of the mitochondrial large subunit and negative regulator of mtDNA transcription.	Cardiomyopathy. Death by 18 weeks.	[89]
Translation Initiation			
**MTIF3**			
***β*** *-actin-cre* homozygous KO		Embryonic lethal.	[2]
*Ckmm-cre* homozygous k KO	MTIF3 regulates the rate of translation initiation, correct mRNA positioning in the preinitiation complex, and removal of prematurely bound initiator tRNA.	Cardiomyopathy.	[2]
*Pf4-cre* homozygous KO	Mitochondrial RNA metabolism is required for platelet activation and thrombus formation.	Thrombocytopenia, increased bleeding time and higher platelet turnover.	[66]
**TACO1**			
Homozygous I164N	Translational activator of COXI through its association with the mitoribosome	Late onset visual impairment, motor dysfunction and cardiac hypertrophy.	[81]
Translation Elongation			
**GFM1**			
Homozygous KO		Embryonic lethal.	[131]
Homozygous R671C		Mild molecular phenotype in liver.	[131]
Compound heterozygous KO/R671C	Models human hepatoencephalopathy due to impaired mitochondrial translation and combined respiratory chain dysfunction.	Hepatoencephalopathy.	[131]
**tRNA** ^**ALA**^			
Mutation	Recapitulates important features of human mtDNA mutation related disease. There are cell-lineage specific mitochondrial gene expression responses to translation dysfunction.	Cardiomyopathy. Mutation selected against in proliferative tissues.	[94, 95]

The genetic tools available in mice are particularly diverse and the advent of CRISPR gene editing has made them even more practical and cost effective to use. Models range from constitutive knockouts or point mutants, to conditional gene modifications that take advantage of the Cre-*loxP* recombination system to precisely control the introduction of mutations at exact times and in specific tissues or cells. Conditional mutants have been particularly advantageous for genes involved in mitochondrial translation as they are typically embryonic lethal, due to their essential roles in energy conversion. Furthermore, conditional mutants enable the investigation of tissue-specific effects of impaired mitochondrial translation, that are often idiosyncratic in these diseases. Inbred mouse lines provide another advantage, as they eliminate the variability between human subjects that can complicate the interpretation of differential disease severity, that is often seen between different human patients with mutations in the same gene. Matched genetic backgrounds also provide the opportunity to model complex traits, such as aging, where the ability to be able to modify a single genetic trait while keeping all other constant is critical. Cross breeding of a large number of different mouse lines with fully-sequenced genomes has recently shown the importance of different genetic backgrounds in modulating physiological phenotypes and provides an additional tool for mouse genetics [157]. In fact, these findings may provide another explanation for certain observed differences between mouse models and human patients—that there are not inherent differences between mice and humans, but rather an effect of epistasis from different gene variants in different cases—such that the examination of genetic mutations in different mouse lines may be an informative approach in future studies.

The provision of new treatments for human diseases requires testing on non-human organisms, before entering further clinical phases. Mouse models provide ideal systems for examining treatment efficacy and potential complications in *in vivo* preclinical studies before testing in patients. Although *in vitro* cell-based models of mitochondrial disease have been valuable in functionally validating disease genes, they are restricted to specific cell types and cannot accurately model the physiological context of diseases, including multi-system interactions or the onset and progression of symptoms in the context of aging. Mouse models, on the other hand, provide a rich resource for preclinical and proof-of-concept evaluation of new disease treatments. This is especially valuable, as potential therapies for mitochondrial diseases are diverse, including nutritional supplements, gene therapies, small molecule drugs, and even environmental interventions. This was highlighted in a recent study that identified the hypoxic response pathway as a modulator of mitochondrial disease [158]. The increased sophistication of mouse models of mitochondrial disease, combined with our advancing understanding of mitochondrial dysfunction, have now set the scene for the development of new treatments and drugs that target impaired mitochondrial translation.
